# Clinical Utility of Serum Autoantibodies Detected by Protein Microarray in Melanoma

**DOI:** 10.1155/2011/413742

**Published:** 2011-10-19

**Authors:** Michael S. Sabel, Yashu Liu, Kent A. Griffith, Jintang He, Xaiolei Xie, David M. Lubman

**Affiliations:** Department of Surgery, University of Michigan Health Systems Biostatistics Core, University of Michigan Comprehensive Cancer Center, Ann Arbor, MI 48109, USA

## Abstract

Better prognostic and predictive markers in melanoma are needed to select patients for therapy. We utilized a dual-lectin affinity chromatography and a natural protein microarray-based analysis to select a subproteome of target glycoproteins to profile serum antibodies against melanoma associated antigens that may predict nodal positivity. We identified 5 melanoma-associated antigens using this microarray coupled to mass spectrometry; GRP75, GRP94, ASAH1, CTSD and LDHB. We evaluated their predictive value for nodal status adjusting for age, gender, Breslow thickness, mitotic rate and ulceration using standard logistic regression. After adjustment, ASAH1, CTSD and LDHB were significantly negatively associated with nodal status (*P* = 0.0008) and GRP94 was significantly positively associated (*P* = 0.014). Our best multivariate model for nodal positivity included Breslow thickness, presence of serum anti-ASAH1, anti-LDHB or anti-CTSD, and presence of serum anti-GRP94, with an area under the ROC curve of 0.869. If validated, these results show promise for selecting clinically node negative patients for SLN biopsy. In addition, there is strong potential for glycoprotein microarray to screen serum autoantibodies that may identify patients at high risk of distant metastases or those likely or unlikely to respond to treatment, and these proteins may serve as targets for intervention.

## 1. Introduction

The present staging system for melanoma, using Breslow thickness, ulceration, mitotic rate, and the presence of regional and distant metastases, stratifies patients into heterogenous groups, with wide variability in outcome or response to therapy. This results in applying more aggressive surgical and adjuvant therapies to large populations, diluting the impact of therapy while exposing more patients to toxicity. Better biomarkers in melanoma are needed to target both surgical and adjuvant therapies, but to date have been elusive. For many solid tumors, the large-scale analysis of gene expression at the RNA level can provide patterns of gene expression that may stratify patients better than TNM staging and help guide therapy. However, this approach requires fresh tissue from a large number of primary tumors, a unique challenge in melanoma where the primary is often only a few millimeters in size, with no residual tissue after the diagnosis has been made. 

For this reason, we chose to examine serum protein markers, hypothesizing that antibody discovery was ideal for the patient with malignant melanoma, as primary tumor tissue is not required and the presence of an immune response to melanoma-associated antigens has been well documented [1–4]. The investigation of humoral response provides new perspective to focus on melanoma-associated antibodies, which are more sensitive and stable to become diagnostic biomarkers for early-stage melanoma. We focused on glycoproteins, as most of the tumor-associated antigens are cell surface proteins or released to the extracellular matrix, where glycosylation is the major type of posttranslational modifications [5, 6]. Moreover, glycoproteins are considered to be the linkage between T cells and antigen-presenting cells to help the orientation of binding, and play important roles in the generation and loading of antigenic peptides into MHC class I and MHC class II [5–7]. 

Using this approach we sought biomarkers that correlated with the presence of regional metastases among melanoma patients. Using dual-lectin affinity chromatography and a natural protein microarray-based analysis to select a subproteome of target glycoproteins which were then used as baits to profile the antibodies against melanoma-associated antigens [8]. This significantly improved technology using lectin affinity chromatography allows us to concentrate low abundant glycoproteins which are typically undetectable in whole cell lysate. This approach led us to the discovery of antibodies to 5 interesting melanoma-associated antigens (75 kD glucose-regulated protein (GRP75), 94 kD glucose-regulated protein (GRP94), acid ceramidase (ASAH1), cathepsin D (CTSD), and lactate dehydrogenase B (LDHB)) that correlated with the presence of melanoma within the regional lymph nodes [8]. GRP75, also known as mortalin, is a transport protein. A member of the heat shock protein-70 family, it also inactivates the tumor suppressor p53. GRP94, also known as heat shock protein-90, is a chaperone protein that is involved in the function and stability of many cell-signaling molecules. ASAH1 is a catabolic lysosomal enzyme that deacylates ceramide, which when phosphorylated forms the poten mitogen S1P. CTSD is a lysosomal acid proteinase which is involved in regulation of programmed cell death. Lactate dehydrogenase (LDH) is an enzyme that catalyzes the conversion of lactate to pyruvate, and serum levels are associated with outcome in stage IV melanoma. We proposed that these autoantibodies may form the basis of a serum test that could select patients for sentinel lymph node biopsy. However, many prognostic factors show limited utility when used clinically in the context of known prognostic factors. We therefore sought to examine the potential clinical utility of these novel serum markers for predicting regional involvement among patients with melanoma.

## 2. Materials and Methods

### 2.1. Patients

In our previous work, we identified serum autoantibodies that recognized glycoproteins from a melanoma cell line and distinguished between 27 node-negative patients and 16 node-positive patients. In that work, we subsequently validated these results using recombinant proteins among a larger sample set of 79 patients. For this University of Michigan Institutional Review Board approved project, we used this latter sample set to examine the clinical utility of these serum autoantibodies as a predictor of regional node involvement. Serum samples were obtained from patients being evaluated at our melanoma multidisciplinary clinic, a few weeks after the diagnostic biopsy, but 2 to 3 weeks prior to undergoing wide local excision and lymph node surgery (SLN biopsy for clinically node-negative patients (*n* = 71) or lymph node dissection for clinically node-positive patients (*n* = 8)). Blood was allowed to clot at room temperature, after which the tubes were centrifugated at 2500 g for 10 minutes. The serum phase was then harvested and frozen in 1 mL aliquots.

### 2.2. Measurement of Serum Autoantibody Levels

Our initial discovery (using extracted glycoproteins) and validation (using recombinant proteins) of these serum autoantibodies have been previously described [8]. For this study we used the results obtained with the recombinant proteins. 75 kD glucose-regulated protein (GRP75), 94 kD glucose-regulated protein (GRP94), cathepsin D (CTSD), and lactate dehydrogenase B (LDHB) were purchased from Abcam (Cambridge, MA, USA). Recombinant acid ceramidase (ASAH1) was purchased from Abnova (Taiwan). These 5 recombinant proteins were chosen because the amino acid sequences described in the manufacturers' instructions are perfectly matched with the sequences acquired from Swiss-Prot database. The sequences and the purity of purchased recombinant proteins were reconfirmed by MALDI-QIT (Shimadzu, CA, USA). 

To summarize our previous work, the recombinant proteins were dissolved in the printing buffer (62.5 mM Tris-HCl (pH 6.8), 1% w/v sodium dodecyl sulfate (SDS), 5% w/v dithiothreitol (DTT), and 1% glycerol in 1x PBS) to reach a final concentration of 100 *μ*g/mL, respectively. Each protein solution was then transferred to a well in a 200 *μ*L 96-well clear printing plate (Bio-rad). The recombinant proteins from the printing plate were spotted onto nitrocellulose (Whatman, USA) slides using a noncontact piezoelectric printer (nanoplotter 2 GeSiM). Each spot contains five spotting events of 500 pL each so that the total volume of each protein solution was 2.5 nL. Each spot was found to be ~450 *μ*m in diameter, with the distance between spots maintained at 600 *μ*m. Printed slides were dried on the printer deck overnight and stored in a refrigerator desiccated at 4°C if the slides were not used immediately. Each recombinant protein was printed in triplicate, and 14 identical blocks were printed on each slide. 

The slides were washed three times with 0.1% Tween-20 in PBS buffer (PBST) and then blocked with 1% bovine serum albumin (Roche) in PBST for 1 hr. The blocked slides were dried by centrifugation and inserted into a SIMplex (Gentel Bioscience) multiarray device which divides each slide by 16 wells. The wells separate the neighboring blocks and prevent cross-contamination. Each serum sample was diluted 1 : 200 in probe buffer which consisted of 1% BSA, 0.05% Triton X-100, 0.1% brij-30 (Sigma-Aldrich, USA) in 1x PBS. The sample hybridization was totally randomized on each slide in no specific order to prevent bias. Each block was hybridized in 100 microliter of diluted serum for 2 hrs at 4°C. Then goat-anti-human IgG (H+L) conjugated with Alexa Fluor 647 (1 *μ*g/mL, Invitrogen, Carlsbad, CA) was applied to each block to bind with the antibodies attached on the protein array. Anti-human IgG was printed on the array as positive control and printing buffer served as the negative control. All processed slides were immediately scanned using an Axon 4000B microarray scanner (Axon Instruments, Foster City, USA). GenePix Pro 6.0 was used to extract the numerical data from each spot on the slides. The background subtracted median intensity of each spot was taken as a single data point. Then the mean intensity of each protein from the triplicate was used for the further analysis.

### 2.3. Statistical Analysis

For this study, patient and tumor characteristics were collected for our sample of 79 melanoma cases, it included patient's age at surgery, gender, tumor thickness (Breslow), mitotic rate, presence of ulceration, and nodal status. For use as a potential clinical test, the sample distribution of each auto-antibody classified as over- or underexpressed, defined as a serum antibody level one standard deviation increment above the sample mean value. Association between the levels of the autoantibodies is summarized by the Spearman rank correlation coefficient with *P* values testing for significant correlations reported. The associations between patient, tumor, and antibody covariates with nodal disease was compared using the two-sample *t*-test for continuous covariates and the chi-square or Fisher's exact test for categorical covariates. The magnitude of the association between each serum antibody level and nodal disease is reported categorically as the odds ratio and 95% confidence interval for cases with overexpression versus cases without. Odds ratios and confidence intervals were reported separately for the univariate associations and after adjustment for the patient and tumor characteristics using standard logistic regression. All statistical analyses were conducted using SAS Version 9.2 (SAS Institute, Inc., Cary, NC, USA) with *P* values less than 5% considered statistically significant.

## 3. Results

**Figure 1:**
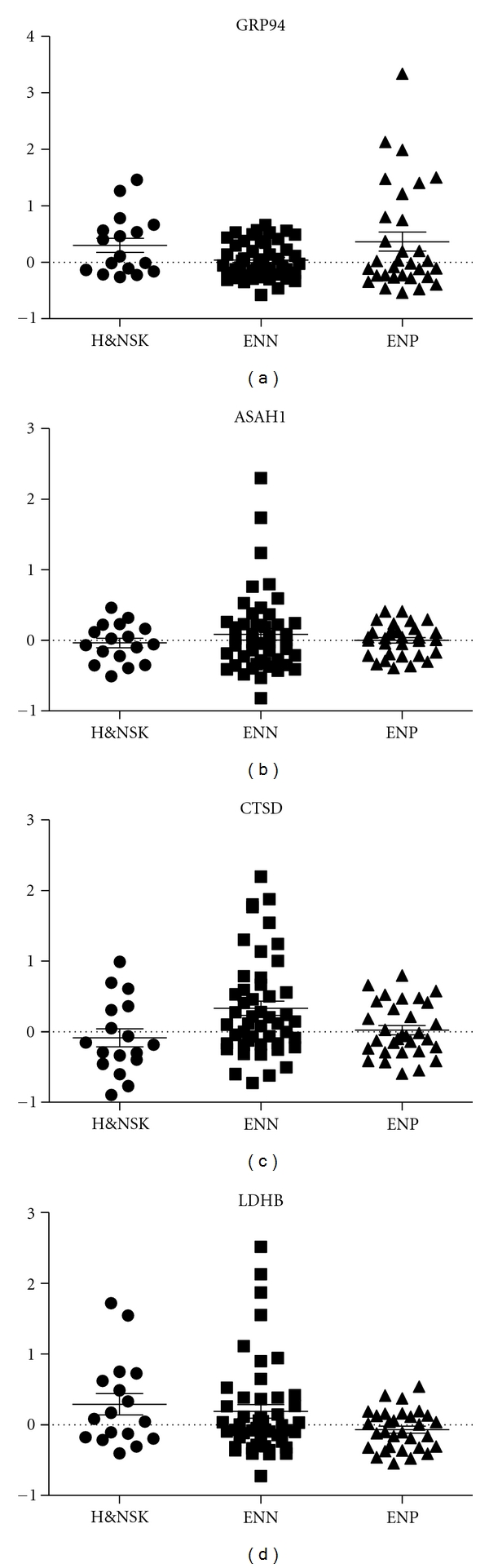
Humoral response to recombinant 75 kD glucose-regulated protein (GRP75), 94 kD glucose-regulated protein (GRP94), acid ceramidase (ASAH1), cathepsin D (CTSD), and lactate dehydrogenase B (LDHB) among healthy volunteers and nonmelanoma skin cancer patients (H&NSK), node-negative melanoma patients (ENN) and node-positive melanoma patients (ENP).

In the previous work, we used the native proteins extracted by a dual-lectin column from the melanoma cell line as bait to detect the presence of autoantibodies in the sera of melanoma patients, identifying 5 antigens including 75 kD glucose-regulated protein (GRP75), 94 kD glucose-regulated protein (GRP94), acid ceramidase (ASAH1), cathepsin D (CTSD), and lactate dehydrogenase B (LDHB), and we investigated the humoral response against the recombinant proteins using a larger sample set of 79 melanoma patients (Figure 1). The clinical characteristics of the patient population are shown in Table 1 for the total population and stratified by nodal disease status. Of note, one patient with a negative SLN subsequently recurred in a regional basin, changing the population from 48 node-negative and 31 node-positive to 47 node-negative and 32 node-positive patients. Among the 32 node-positive patients, 8 were clinically node-positive, 23 were SLN positive, and 1 represented a regional recurrence after false-negative SLN. Each glycoprotein was summarized by over- (>1SD), standard (±1SD), and underexpression (<1SD), relative to the sample mean. ASAH1, CTSD, and LDHB all had significant negative associations with the presence of nodal disease, with overexpression associated with a lower risk. Higher GRP94 levels were associated with a higher risk of nodal disease; however, the level of GRP75 was not significantly associated with nodal status. Correlation between antibody levels and age, gender, Breslow thickness, mitotic rate, or ulceration are shown in Table 2. Correlations included ASAH1 which negatively correlated with patient age (although the magnitude of the correlation was mild, *r* < 0.3) and GRP75 which was higher in females than males (*P* = 0.03).

**Figure 2:**
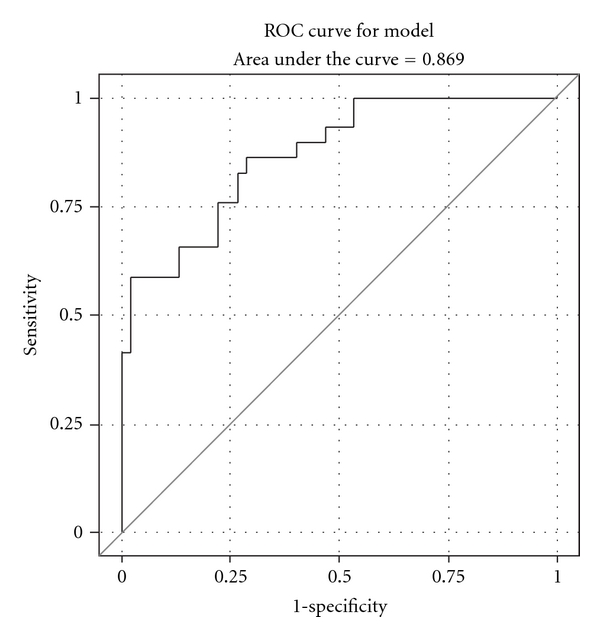
Receiver operating characteristic (ROC) curve for our best multivariable model for nodal positivity incorporating increasing Breslow thickness (increased risk), autoantibody response to GRP94 (increased risk), and autoantibody response to ASAH1, LDHB, or CTSD (decreased risk).

The magnitude of the association of the glycoproteins with nodal disease status is reported in Table 3. Even after adjustment for clinical parameters (age, gender, ulceration, and Breslow thickness) ASAH1, CTSD, and LDHB remained significantly negatively associated with nodal disease, and GRP94 positively associated (Table 4). A composite measure for the overexpression of any of the three proteins shown to have a negative association with nodal disease (ASAH1, CTSD, and LDHB) was constructed with 22 (27.9%) of the total population with composite overexpression. Only 2 (6.2%) of the node-positive patients had composite overexpression in contrast to 20 (42.5%) node-negative patients. For the 9 of 32 node-positive patients (28%) who had overexpression of GRP94, 5 (16%) patients had clinically involved nodes while the remaining 4 (14%) had microscopic disease (<2% surface area) in clinically negative nodes. Table 5 reports the best multivariable model for nodal positivity. Overexpression of anti-ASAH1, anti-LDHB, or anti-CTSD (decreased risk), the overexpression of anti-GRP94 (increased risk) and Breslow thickness (increased risk) significantly correlated with the likelihood of regional metastases. The ROC curve for this model is presented in Figure 2, with an area under the curve of 0.8690.

## 4. Discussion

Using dual-lectin affinity chromatography to generate a subproteome of glycoproteins from a melanoma cell line generated from a metastatic deposit, we discovered 4 antibodies in the serum of melanoma patients recently diagnosed with melanoma that were strongly correlated with the presence or absence of nodal metastases. In this analysis, we demonstrate that overexpression of these antibodies were independent of other known prognostic factors in melanoma, and on multivariate analysis maintained highly significant, independent prognostic value. These results demonstrate the potential of these 4 autoantibodies as a serum test for the purpose of selecting clinically node-negative patients for SLN biopsy. Elevation (defined as >1 SD above the mean) of anti-ASAH1, anti-CTSD, or anti-LDHB was highly significantly associated with being SLN negative, with an odds ratio of 0.05 (0.01–0.31, *P* = 0.002) after adjusting for age, gender, ulceration, mitotic rate, and Breslow thickness. Elevation of one of these three antibodies was not uncommon; among the 70 clinically node-negative patients in this study, 22 patients (33%) had elevation of one of these antibodies. If it were validated that patients with elevation of one of these antibodies had a very low risk of SLN metastases, then potentially these patients could be treated by wide excision alone, reducing the cost and morbidity of melanoma treatment. In contrast, elevation of anti-GRP94 was associated with an increased risk of regional metastases, and was elevated in both patients with clinically evident disease (5 of 8 patients) and microscopic disease (4 of 23 patients). While detection of serum anti-GRP94 levels would be less useful clinically, it could potentially identify some patients with thin melanoma for whom SLN might otherwise be omitted. 

A bigger question is the role these proteins may play in the development and progrsion of melanoma. Beyond the development of serum-based diagnostic tests, proteomics may identify targets for therapeutic intervention. Indeed, 3 of the 4 proteins have strong associations with melanoma progression and prognosis. Lactate dehydrogenase (LDH) is an enzyme that catalyzes the conversion of lactate to pyruvate, and serum levels of LDH are strongly associated with melanoma prognosis. Serum LDH levels strongly correlate with outcome among stage IV patients and serum LDH measurements are part of the American Joint Cancer Commission (AJCC) staging system for melanoma [9–13]. However, serum LDH levels are rarely elevated and of no clinical utility in nonmetastatic melanoma [14–16]. Cathepsin D (CTSD) is a lysosomal acid proteinase which degrades proteins, peptides, and peptide precursors. In addition, it appears to be involved in other biological processes including regulation of programmed cell death, tissue remodeling and renewal, activation of proteolytic enzymes, and fibrinolysis [17]. Many tumors have altered processing, secretion, and activity levels of CTSD, and they are often associated with aggressive behavior, stimulating tumor cell proliferation, invasion, and metastases [17, 18]. Immunohistochemical studies have shown that CTSD is markedly expressed in melanoma cell lines and tissue biopsies from primary and metastatic melanoma, and these correlate with poor outcome [19–24]. As with LDH, measuring plasma levels of CTSD was not of clinical value for identifying patients at risk of recurrence [17, 25]. As we can detect very low levels of antibodies in the serum, measuring antibody levels may be more sensitive than measuring protein levels, allowing transition of known serum markers from utility in stage IV disease only to the setting of early-stage disease. Prior to this publication, the third protein, acid ceramidase (ASAH1), had not been strongly associated with melanoma progression, but has been associated with cancers of the breast, prostate, and thyroid [26–29]. ASAH1 is a catabolic lysosomal enzyme that deacylates ceramide and yields sphingosine, which when phosphorylated, forms the potent mitogen S1P. The cellular levels of ceramide, sphingosine, and S1P are integral in determining cell survival and growth [30–32]. Targeting this pathway holds promise for anticancer therapies [32–34]. 

In the case of these three proteins, expression of the proteins is associated with advanced stage, but the presence of antibodies to these proteins is associated with lower stage. This highlights one drawback to antibody-array-based proteomics—the presence of the antibody may be due to increased exposure of the proteins (overexpression), immune recognition of protein alteration, or the antibodies may be functionally blocking critical pathways. In the case of these three proteins/antibodies, it remains unclear whether this represents specific functional inhibition by the antibodies, or increased progression in the face of decreased immune recognition of overexpressed proteins (disease advancement in the face of decreased immune surveillance). As these proteins are identified, further analysis of their role in melanoma progression, and their posttranslational structure is necessary.

In contrast to these three proteins, for which the detection of autoantibodies was a favorable prognostic sign, the presence of autoantibodies to GRP94, or heat shock protein-90 (HSP90), was associated with an increased risk of regional metastases. Although this protein is highly conserved (and should not trigger a significant immune response), our primary data and validation studies using the recombinant proteins demonstrate the presence of anti-GRP94 antibodies in the serum of nearly between 1/4 and 1/3 of node-positive patients. HSP90 is a chaperone protein that is crucially involved in the function and stability of many oncogene products and cell-signaling molecules, including CRAF, ERB-B2, BCR-ABL, CDK4, CDK6, AKT, mutated p53, MEK, VEGFR, and importantly to melanoma mutated (but not wildtype) BRAF. HSP90 protects these proteins from deterioration caused by environmental stress, which includes cancer therapy. Expression of HSP90 is elevated in melanoma, correlates with increasing Breslow thickness, and is associated with advanced disease [35]. Because HSP90 chaperones so many proteins implicated in carcinogenesis, inhibiting HSP90 could inhibit several pathways at once, HSP90 inhibitors are presently in clinical trial in metastatic melanoma. While our data suggest that autoantibodies do little to inhibit HSP90 functionally, their presence as a response to overexpression is clearly related to melanoma progression.

In conclusion, the creation of a glycoprotein microarray to screen melanoma patient serum samples for autoantibodies yielded four autoantibodies that show promise in predicting regional metastases, and could potentially form the basis of a blood test to select clinically node-negative patients for SLN biopsy. If validated, this test could greatly minimize the cost and morbidity associated with the surgical treatment of melanoma, as well as identify patients with thin melanomas who should undergo the procedure. In addition, glycoproteins recognized by these antibodies may have important roles in the development and progression of melanoma and may serve as targets for intervention. On a broader scale, this approach could be used to identify additional serum autoantibodies that can identify patients at high risk of distant metastases and those unlikely to respond to treatment, allowing a more tailored use of adjuvant therapies.

## Figures and Tables

**Table 1:** Patient and tumor characteristics of the study cohort.

Characteristic	Total	Node negative	Node positive	*P* value^1^
Frequency	79	47	32	

Age				
Mean (SD)	51.9 (12.6)	51.8 (13.2)	52.0 (12.0)	0.944

Gender				
Males: *N* (%)	38 (48.1)	22 (46.8)	16 (50.0)	0.781
Females: *N* (%)	41 (51.9)	25 (53.2)	16 (50.0)	

Breslow depth				
Mean (SD)	2.39 (1.65)	1.94 (1.15)	3.11 (2.04)	0.007

Mitotic rate				
Mean (SD)	5.51 (5.72)	4.45 (4.93)	7.04 (6.50)	0.075

Ulceration				
Absent: *N* (%)	24 (30.4)	35 (74.5)	16 (50.0)	0.059^2^
Present: *N* (%)	51 (64.6)	11 (23.4)	13 (40.6)	
Unknown: *N* (%)	4 (5.0)	1 (2.1)	3 (9.4)	

ASAH1				
Mean (SD)	803 (353)	837 (432)	753 (179)	0.238
±1 SD: *N* (%)	68 (86.1)	36 (76.6)	32 (100)	0.008
>1 SD: *N* (%)	8 (10.1)	8 (17.0)	0	
<1 SD: *N* (%)	3 (3.8)	3 (6.4)	0	

CTSD				
Mean (SD)	8664 (4355)	9607 (5014)	7278 (2660)	0.009
±1 SD: *N* (%)	62 (78.5)	33 (70.2)	29 (90.6)	0.039
>1 SD: *N* (%)	12 (15.2)	11 (23.4)	1 (3.1)	
<1 SD: *N* (%)	5 (6.3)	3 (6.4)	2 (6.3)	

GRP75				
Mean (SD)	4087 (2107)	4221 (2367)	3891 (1671)	0.471
±1 SD: *N* (%)	62 (78.5)	36 (76.6)	26 (81.3)	0.913
>1 SD: *N* (%)	13 (16.5)	8 (17.0)	5 (15.6)	
<1 SD: *N* (%)	4 (5.1)	3 (6.4)	1 (3.1)	

GRP94				
Mean (SD)	6798 (3782)	6032 (1840)	7924 (5364)	0.063
±1 SD: *N* (%)	70 (88.6)	47 (100)	23 (71.9)	<0.001
>1 SD: *N* (%)	9 (11.4)	0	9 (28.1)	
<1 SD: *N* (%)	0	0	0	

LDH				
Mean (SD)	7863 (4093)	8587 (4933)	6798 (2021)	0.029
±1 SD: *N* (%)	68 (86.1)	37 (78.7)	31 (96.9)	0.056
>1 SD: *N* (%)	10 (12.7)	9 (19.2)	1 (3.1)	
<1 SD: *N* (%)	1 (1.3)	1 (2.1)	0	

Combination: ASAH1, CTSD, and LDH				
Overexpressed^†^	22 (27.9)	20 (42.5)	2 (6.2)	<0.001
Normal	57 (72.1)	27 (57.5)	30 (93.8)	

^1^Comparing between node-negative and node-positive groups.

^2^Unknown group omitted for statistical test.

^†^Overexpressed defined as >1 SD for ASAH1, CTSD, or LDH.

**Table 2:** Correlation of antibodies and continuous patient and tumor characteristics: Spearman *r*, *P* value.

	ASAH1	CTSD	GRP75	GRP94	LDH
Age	**−0.2317**	**0.0811**	**−0.0024**	**0.0214**	**0.0539**
	0.0400	0.4774	0.9836	0.8514	0.6371
Breslow	**0.1294**	**0.0822**	**0.1975**	**0.0878**	**0.1444**
	0.2686	0.4834	0.0894	0.4539	0.2165
Mitotic rate	**0.0630**	**−0.0253**	**0.0516**	**0.0705**	**0.0485**
	0.6019	0.8341	0.6693	0.5593	0.6881

**Table 3:** Univariate associations of glycoproteins with positive nodal status.

Characteristic	Odds ratio	95% CI	*P* value
ASAH1			
500 unit increase	0.685	0.332–1.415	0.306
>1 SD versus not	0.123^†^		0.019
CTSD			
500 unit increase	0.928	0.869–0.991	0.026
>1 SD versus not	0.106	0.013–0.864	0.022
GRP75			
500 unit increase	0.962	0.860–1.076	0.495
>1 SD versus not	0.903	0.266–3.059	0.999
GRP94			
500 unit increase	1.076	1.002–1.156	0.0440
>1 SD versus not	38.4^†^		<0.0001
LDH			
500 unit increase	0.931	0.862–1.007	0.0731
>1 SD versus not	0.193^†^		0.0427
Combination: ASAH1, CTSD, and LDH			
Any >1 SD versus not	0.173	0.045–0.663	<0.0001

^†^Continuity correction applied when calculating the estimate of odds ratios due to cell sample sizes ≤1. Confidence interval not reportable in these cases.

**Table 4:** Adjusted^†^ associations of glycoproteins with positive nodal status.

Characteristic	Odds ratio	95% CI	*P* value
ASAH1			
500 unit increase	0.272	0.076–0.979	0.0464
>1 SD versus not	Model not estimable		
CTSD			
500 unit increase	0.867	0.786–0.956	0.0042
>1 SD versus not	0.067	0.006–0.740	0.0275
GRP75			
500 unit increase	0.880	0.749–1.034	0.1216
>1 SD versus not	0.644	0.153–2.712	0.5488
GRP94			
500 unit increase	1.052	0.976–1.133	0.1871
>1 SD versus not	Model not estimable		
LDH			
500 unit increase	0.890	0.797–0.993	0.0363
>1 SD versus not	0.135	0.015–1.210	0.0736
Combination: ASAH1, CTSD, and LDH			
Any >1 SD versus none	0.045	0.007–0.309	0.0016

^†^Adjusted for age, gender, ulceration, and Breslow depth. Due to the very high correlation between mitotic rate and Breslow depth, mitotic rate was omitted from the model.

**Table 5:** Best multivariable model explaining positive nodal status.

Characteristic	Odds ratio	95% CI	*P* value
Age			
1 year increase	0.987	0.935–1.043	0.6468
Gender			
Male	1.663	0.426–6.495	0.4645
Female	1.000		
Ulceration			
Present	2.046	0.359–11.662	0.4201
Absent	1.000		
Breslow			
1 mm increase	2.178	1.104–4.298	0.0248
Combination: ASAH1, CTSD, and LDH			
Overexpressed	0.006	< 0.001–0.117	0.0008
Not overexpressed	1.000		
GRP94			
Overexpressed	1.223	1.041–1.436	0.0141
Not overexpressed	1.000		
